# Diagnosis and Treatment of Autoimmune Pancreatitis in China: A Systematic Review

**DOI:** 10.1371/journal.pone.0130466

**Published:** 2015-06-25

**Authors:** Qianqian Meng, Lei Xin, Wenyu Liu, Han Lin, Bo Tian, Luowei Wang, Zhaoshen Li

**Affiliations:** 1 Department of Gastroenterology, Changhai Hospital, Second Military Medical University, Shanghai, China; 2 Department of General Surgery, Changhai Hospital, Second Military Medical University, Shanghai, China; University of Szeged, HUNGARY

## Abstract

**Aims:**

To provide comprehensive data on the diagnosis and treatment of autoimmune pancreatitis (AIP) patients in China.

**Design:**

A systematic review.

**Methods:**

All clinical studies concerning AIP from China published between January 2006 and June 2014 were retrospectively reviewed and analyzed.

**Results:**

A total of 26 original articles involving 706 AIP patients were included with an estimated proportion of type 2 AIP as 4.7%. In the 706 AIP patients, the range of mean/median age was 48.6–67.0 years old and the male to female ratio was 4.47:1. The common presentations included obstructive jaundice (pooled rate: 63.4%, 95%CI: 55.4%–71.0%) and abdominal symptoms (pooled rate: 62.3%, 95%CI: 52.4%–71.7%). Biliary involvement was the most common extrapancreatic manifestations, especially the lower part of the common bile duct (pooled rate: 62.3%, 95%CI: 49.9%–73.9%). According to the imaging examinations, 53.8% and 41.6% patients were classified into focal-type and diffuse-type, respectively. Notably, upstream pancreatic duct dilatation was found in parts of patients (pooled rate: 13.8%, 95%CI: 6.6%–23.1%). The levels of serum IgG4 were elevated in most patients (pooled rate: 86.0%, 95%CI: 74.2%–94.6%). Nearly three tenths AIP patients received surgery (pooled rate: 29.7%, 95%CI: 18.1%–42.8%) due to mimicked malignancy. Steroid treatment was given to 78.4% patients (95%CI: 65.3%–89.1%) with a pooled remission rate of 96.2% (95%CI: 94.0%–97.9%). The pooled relapse rate was 13.8% (95%CI: 7.2%–22.0%) with the mean follow-up time ranging from 12 to 45 months.

**Conclusion:**

Type 1 is the predominant type of Chinese AIP patients and the clinical features, diagnostic modalities and therapeutic regimen were similar with those in other countries. Knowledge of AIP should be more widespread to avoid unnecessary surgery.

## Introduction

Since it was first reported in 1961 by Sarles *et al* [1] and was termed as autoimmune pancreatitis (AIP) in 1995 by Yoshida *et al* [2], AIP has gradually attracted attention in recent years. Nowadays, two subtypes of AIP are known—type 1 is a multi-organ disease associated with IgG4 while type 2 appears to be a pancreas-specific disorder [3]. In recent years, diagnostic criteria of AIP have been proposed in Japan, United States, Korea and Italy, and an International Consensus Diagnostic Criteria (ICDC) for AIP has been reached [4].

China has a high prevalence of pancreatic disease [5], and if the prevalence rate of AIP in Japan (2.2 per 100,000 populations) was used, the estimated amount of AIP patients would be 29 thousand [6]. However, there have been only a few reports about AIP from China [7–10]. In this study, we collected all well-designed clinical studies of AIP from China, and provided comprehensive data on the clinical presentation, diagnosis and treatment of Chinese AIP patients.

## Methods

### Strategy, criteria, and procedures for the literature search

We conducted a systematic literature search of PubMed, Web of Science and Chinese literature database including Wanfang Data and CNKI on June 20, 2014, and all publications related to AIP between January 2006 and June 2014 were retrieved. Indexing terms used for searching were autoimmune pancreatitis, IgG4-related disease, lymphoplasmacytic sclerosing pancreatitis, idiopathic duct centric pancreatitis, chronic pancreatitis with autoimmune diseases. Inclusion and exclusion criteria were delineated before commencement of the literature search. Inclusion criteria included: (1) cases were Chinese patients; (2) the diagnostic criteria of AIP were clearly defined and consistent with the current accepted ones, which included ICDC [4] and criteria proposed by Japan [11, 12], Mayo Clinic [13], Korea [14], and Asian (Korea-Japan symposium) [15]; (3) The number of patients in single article is ≥ 10; (4) if multiple articles were from the same institution, the one with most patients were selected. All articles other than original contributions, such as case reports, reviews, meta-analyses, editorials and letters were excluded. This systematic review was performed in accordance with the Preferred Reporting Items for Systematic Reviews and Meta-Analyses (PRISMA) statement checklist (S1 PRISMA Checklist).

### Definitions

In order to include all relevant articles without loss of the fundamental meanings of the terms, clinical presentation and extrapancreatic manifestation were defined in a relatively broad way. Abdominal symptoms included abdominal pain or discomfort. Because the diagnostic criteria for acute pancreatitis were not clearly given in most of the literature, the presentation of acute pancreatitis mentioned in some articles was classified as abdominal symptoms. Bile duct involvement means bile duct stricture, divided into lower part of the common bile duct stricture and hilar/intrahepatic bile ducts stricture. Because ICDC clearly defines the subtypes of AIP, the proportion of type 1 and type 2 AIP was estimated according to studies adopting ICDC as criteria.

### Data extraction from eligible articles

Data on the clinical presentations, laboratory tests, imaging examinations, pathological examinations, treatment and outcomes of the patients were retrieved. Missing data or indeterminate definitions were resolved by direct contact with authors if possible and were otherwise considered not available. Two authors (Q.Q.M., L.X.) determined the relevant original articles and extracted the data independently, whereas the third author (W.Y.L) checked the results. If a disagreement existed, the relevant programs were repeated until a consensus was achieved among the authors.

### Statistical analysis

Descriptive analysis or meta-analysis was conducted to provide the overall data of clinical presentation, laboratory test, imaging examination, pathological examination, treatment and outcome of the patients. In the meta-analysis, Cochran’s Q test was used to test the heterogeneity of results across studies, and I^2^ was calculated to quantify the heterogeneity among studies. If the p value was less than 0.10 (Cochran’s Q test), the results were considered heterogeneous and random-effects model was used to obtain the pooled estimates. Otherwise, fixed-effects model was applied. The potential presence of publication bias was evaluated statistically by the Begg-Mazumdar’s and Egger’s test. Meta-analysis was performed with StatsDirect Statistical software, version 2.7.8 (StatsDirect Ltd, Altrincham, UK).

## Results

**Fig 1:**
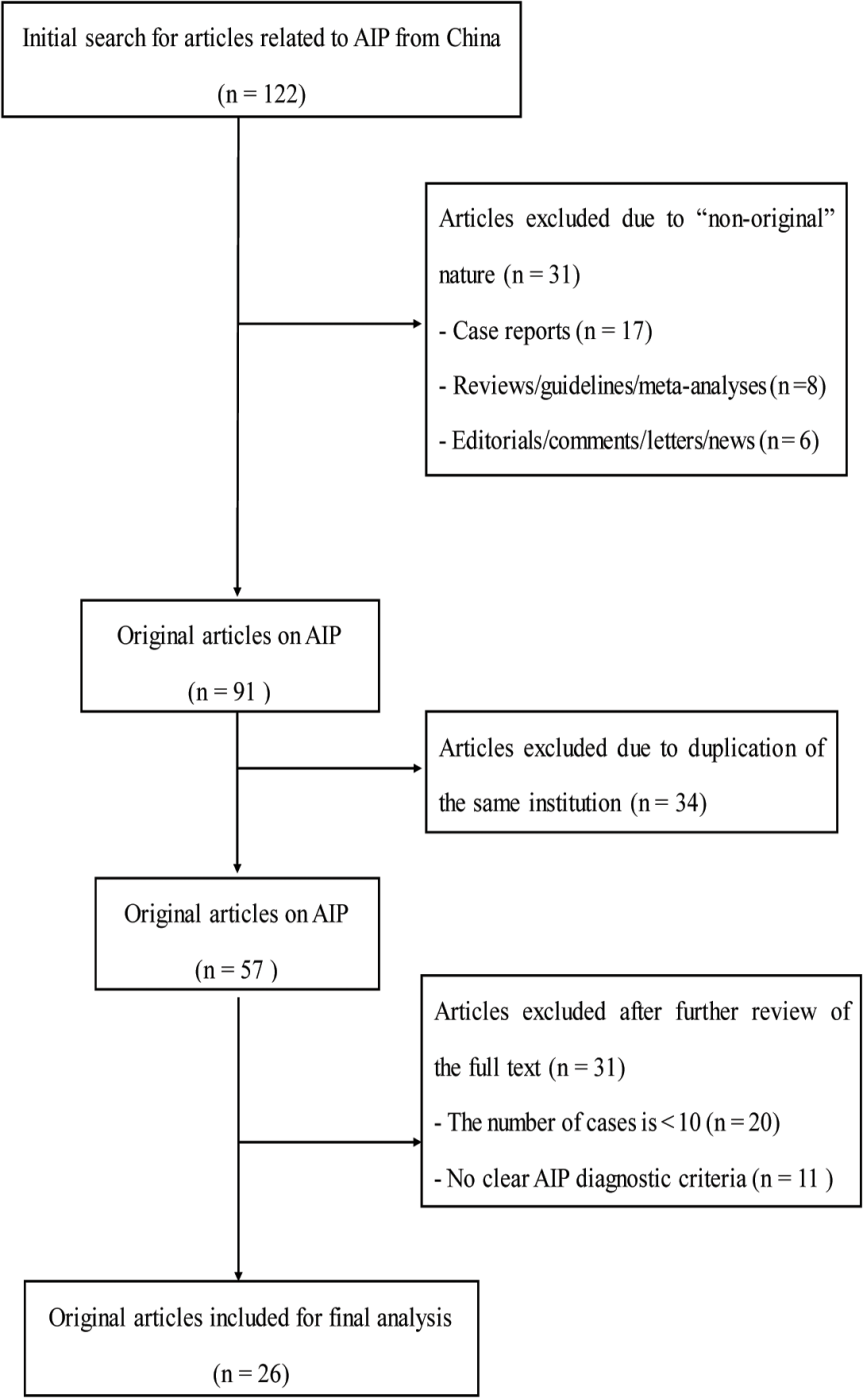
Flow chart for literature search on original articles about Chinese AIP patients.

A total of 26 articles (5 English-language and 21 Chinese-language articles) were enrolled in the study ([8, 9, 16] and S1 File). The detailed search procedures are outlined in *Fig 1*. The detailed data related to diagnostic criteria for AIP are outlined in Table 1. In the 5 studies which adopted ICDC as criteria, the proportion of type 2 AIP was 4.7% (8/171). Detailed data and forest plots for all of the variables are listed in S2 File.

**Table 1:** The diagnostic criteria for AIP adopted by 26 studies from China (N = 706)^a^.

Diagnostic Criteria	No.of studies (%)	No. of cases (%)
Japanese criteria	7 (26.9)	126 (17.8)
Mayo Clinic's HISORt criteria	3 (11.5)	150 (21.2)
Korean criteria	2 (7.7)	30 (4.2)
Asian diagnostic criteria	12(46.2)	416(58.9)
ICDC	5(19.2)	171(24.2)

^a^ Different kinds of diagnostic criteria for AIP were adopted by three studies

### Demographics and Clinical Presentation

Among the 706 AIP patients, 577 were men (male/female: 4.47:1) and the range of mean/median age of the patients was 48.6–67.0 years old.

Clinical presentation of AIP patients were described in all 26 articles. Obstructive jaundice is the most common presentation (pooled rate: 63.4%, 95%CI: 55.4%–71.0%). Abdominal symptoms was another common presentation (pooled rate: 62.3%, 95%CI: 52.4%–71.7%). The other presentations included weight loss (pooled rate: 45.1%, 95%CI: 34.8%–55.6%) and diabetes mellitus (pooled rate: 26.9%, 95%CI: 18.3%–36.4%). Moreover, pancreatic mass or enlargement was found incidentally by imaging examinations in some asymptomatic patients (pooled rate: 2.9%, 95%CI: 1.6%–4.5%) (Table 2).

**Table 2:** Pooled positive rate of various manifestation in Chinese AIP patients.

				Tests of Heterogeneity			Tests of Publication Bias	
Manifestation	Studies No.	Positive Cases / Total Cases	Pooled Positive Rate, % (95%CI)	Q Value	P	I ^2^ (%)	Begg’s P	Egger’s P
Clinical presentation								
Obstructive jaundice	26	432/706	63.4(55.4–71.0)	116.89	<0.0001	78.6	0.1932	0.0185
Abdominal symptoms	26	382/706	62.3(52.4–71.7)	178.82	<0.0001	86	0.3655	0.8228
Weight loss	18	206/495	45.1(34.8–55.6)	94.18	<0.0001	81.9	0.2107	0.0008
Diabetes mellitus	23	157/595	26.9(18.3–36.4)	139.53	<0.0001	84.2	0.4918	<0.0001
Asymptomatic patients	26	22/706	2.9(1.6–4.5)	34.23	0.103	27	<0.0001	0.1127
Extrapancreatic manifestation								
Lower part of the common bile duct stricture	19	290/473	62.3(49.9–73.9)	131.11	<0.0001	86.3	0.7792	0.0086
Hilar/intra hepatic bile ducts stricture	17	32/409	3.6(1.1–7.3)	45.66	0.0001	65	0.0063	0.7545
Abdominal lymph nodes enlargement	20	99/420	19.7(9.2–32.9)	190.81	<0.0001	90	0.0007	0.0139
Swelling salivary gland	18	51/466	12.0(5.7–20.3)	98.38	<0.0001	82.7	<0.0001	0.0022
Swelling lacrimal gland	18	12/504	2.7(1.0–5.1)	32.33	0.0137	47.4	<0.0001	0.0165
Symptom similar with Sjögren syndrome	12	26/274	8.9(4.2–15.1)	29.24	0.0021	62.4	0.0088	0.0038
Interstitial nephritis	18	29/515	4.6(1.9–8.5)	52.57	<0.0001	67.7	0.0003	0.0361
Retroperitoneal fibrosis	14	15/464	3.4(2.0–5.2)	16.84	0.2066	22.8	0.0546	0.1481
Interstitial lung disease	16	15/471	3.0(1.0–6.0)	36.92	0.0013	59.4	0.0001	0.0844
Ulcerative colitis	14	4/446	1.6(0.6–2.9)	4.19	0.989	0	0.0748	0.7726

The details of extrapancreatic manifestation were reported in 20 articles. Biliary involvement was the most common extrapancreatic manifestation, which included lower part of the common bile duct stricture (pooled rate: 62.3%, 95%CI: 49.9%–73.9%) and hilar/intra hepatic bile ducts stricture (pooled rate: 3.6%, 95%CI: 1.1%–7.3%). Abdominal lymph nodes enlargement is a relatively common manifestation (pooled rate: 19.7%, 95%CI: 9.2%–32.9%). Fifty-one (pooled rate: 12.0%, 95%CI: 5.7%–20.3%) patients and 12 (pooled rate: 2.7%, 95%CI: 1.0%–5.1%) patients have swelling salivary gland and swelling lacrimal gland, respectively, while 26 (pooled rate: 8.9%, 95%CI: 4.2%–15.1%) patients have symptom similar with Sjögren syndrome. Other extrapancreatic manifestations included interstitial nephritis (pooled rate: 4.6%, 95%CI: 1.9%–8.5%), retroperitoneal fibrosis (pooled rate: 3.4%, 95%CI: 2.0%–5.2%), interstitial lung disease (pooled rate: 3.0%, 95%CI: 1.0%–6.0%) and ulcerative colitis (pooled rate: 1.6%, 95%CI: 0.6%–2.9%) (Table 2).

### Imaging examinations

The general manifestation of pre-treatment computed tomography (CT), magnetic resonance imaging (MRI) or ultrasound examination was reported in 627 patients from 25 articles. Among these 627 patients, 337 (53.8%) were classified into diffuse-type, 261 (41.6%) were focal-type and 29 (4.6%) with atypical imaging manifestation could not be clearly classified into either type. Most lesions of focal-type were located in the head (74.4%, 174/234), and the other were located in the body (11.5%, 27/234) and the tail (14.1%, 33/234). Delayed enhancement was the most typical manifestation (272/286, pooled rate: 94.4%, 95%CI: 88.5%–98.2%). Moreover, rim-like enhancement is found in considerable part of patients (226/445, pooled rate: 62.7%, 95%CI: 48.3%–76.0%). Calcification in parenchyma (18/487, pooled rate: 3.9%, 95%CI: 1.8%–6.8%) and pancreatic pseudocysts (14/487, pooled rate: 3.4%, 95%CI: 2.0%–5.1%) were rare manifestations (Table 3).

**Table 3:** Pooled positive rate of various imaging manifestations in Chinese AIP patients.

				Tests of Heterogeneity			Tests of Publication Bias	
Imaging Manifestation	Studies No.	Positive Cases/ Total Cases	Pooled Positive Rate, % (95%CI)	Q Value	P	I ^2^ (%)	Begg’s P	Egger’s P
Parenchymal imaging								
Delayed enhancement	13	272/286	94.4 (88.5–98.2)	38.41	0.0001	68.8	<0.0001	0.0018
Rim-like enhancement	18	226/445	62.7 (48.3–76.0)	162.47	<0.0001	89.5	0.3686	0.5849
Calcification in parenchyma	21	18/487	3.9 (1.8–6.8)	39.94	0.0051	49.9	<0.0001	0.0033
Pancreatic pseudocysts	21	14/487	3.4 (2.0–5.1)	17.38	0.6281	0	0.0303	0.0394
Ductal imaging								
Stricture of the pancreatic duct	18	231/347	68.1 (49.3–84.3)	232.83	<0.0001	92.7	0.0813	0.0035
Dilation of pancreatic duct	19	64/367	13.8 (6.6–23.1)	101.00	<0.0001	82.2	<0.0001	0.0007
Pancreatic duct calculi	20	6/460	1.9 (0.9–3.3)	11.97	0.8869	0	<0.0001	0.2087

In the aspect of pancreatic duct, about two-thirds patients showed stricture of pancreatic duct (231/347, pooled rate: 68.1%, 95%CI: 49.3%–84.3%) and dilatation was also found in parts of patients (64/367, pooled rate: 13.8%, 95%CI: 6.6%–23.1%). Pancreatic duct calculi was rare manifestation (6/460, pooled rate: 1.9%, 95%CI: 0.9%–3.3%) (Table 3).

### Laboratory tests

Various serum markers were measured in different amount of patients (Table 4). In 9 studies, the level of serum IgG4 exceeds the upper limit of normal in 170 of 195 patients (pooled rate: 86.0%, 95%CI: 74.2%–94.6%). The positive rates of IgG and IgE were 65.7% (257/391, pooled rate: 70.2%, 95%CI: 60.6%–79.1%) and 43.4% (49/113, pooled rate: 61.8%, 95%CI: 23.7%–92.9%), respectively.

**Table 4:** Pooled positive rate of various serum markers in Chinese AIP patients.

				Tests of Heterogeneity			Tests of Publication Bias	
Serum markers	Studies No	Positive Cases / Total Cases	Pooled Positive Rate, % (95%CI)	Q Value	P	I ^2^(%)	Begg’s P	Egger’s P
IgG4 ^**a**^	9	170/195	86.0(74.2–94.6)	31.71	0.0001	74.8	0.0446	0.0265
IgG ^**b**^	19	257/391	70.3(60.6–79.1)	74.87	<0.0001	76	0.195	0.3595
IgE ^**c**^	4	49/113	61.8(23.7–92.9)	37.6	<0.0001	91.9	0.75	0.7564
γ-globulin	12	154/225	67.9(56.9–78.0)	34.67	0.0003	68.3	0.0138	0.0019
Rheumatoid factor	6	34/110	35.7(20.7–52.2)	14.75	0.0115	66.1	0.2722	0.0517
ANA ^**d**^	13	83/266	33.3(24.8–42.4)	29.21	0.0037	58.9	0.0573	0.074
ESR ^**e**^	7	65/109	59.4(50.4–68.2)	10.20	0.1163	41.2	0.1668	0.0051
CA19-9	18	178/411	41.5(34.9–48.3)	32.16	0.0144	47.1	0.9697	0.3708

^**a**^Immunoglobulin G4

^**b**^Immunoglobulin G

^**c**^Immunoglobulin E

A

^**d**^Anti-neutrophil antibody

^**e**^Erythrocyte sedimentation rate

### Pathology

In the 706 patients, pancreatic cytology specimens were obtained in 79 (11.2%) by endoscopic ultrasonography (EUS)-guided or CT-guided fine-needle aspiration (FNA) and no malignant or atypical cells were found. Pancreatic histology specimens were obtained from 220 (31.2%) patients by surgery. A total of 212 and 8 histology examinations supported the diagnosis of lymphoplasmacytic sclerosing pancreatitis and idiopathic duct-centric pancreatitis respectively. In the 66 patients (4 studies) receiving pancreatic immunohistochemical examination, marked IgG4 positive cells (>10 cells / high-power fields [HPF]) were found in 58 (pooled rate: 88.7%, 95%CI: 65.2%–99.7%) patients.

### Treatment and follow-up

Due to mimicked malignancy, nearly one third AIP patients received surgery (pooled rate: 29.7%, 95%CI: 18.1%–42.8%). Surgical procedures included pancreaticoduodenectomy (43.0%, 52/121), distal pancreatectomy with or without splenectomy (9.1%, 11/121), choledojejunostomy with the resection of hilar mass (1.6%, 2/121), biliary-entericanastomosis (27.3%, 33/121) and exploratory laparotomy (19.0%, 23/121).

Steroid treatment was given to most patients (pooled rate: 78.4%, 95%CI: 65.3%–89.1%). In the 272 patients with detailed regimen, a fixed dose (30mg or 40mg prednisone per day) were given as the initial dose to 206 (75.7%) patients, while a personalized initial dose (0.6mg/kg per day) were given to other 66 (24.3%) patients. After steroid therapy, most patients got remission (pooled rate: 96.2%, 95%CI: 94.0%–97.9%). In 269 patients receiving steroid treatment and having follow-up data, relapse occurred in 37 patients (pooled rate: 13.8%, 95%CI: 7.2%–22.0%) with the mean follow-up time ranging from 12 to 45 months. Repeated steroid treatment was given to most patients with relapse (pooled rate: 95.8%, 95%CI: 89.8%–99.3%) and obtained good remission outcome (pooled rate: 94.8%, 95%CI: 88.2%–98.8%) (Table 5).

**Table 5:** Pooled positive rate of treatment and prognosis in Chinese AIP patients.

				Tests of Heterogeneity			Tests of Publication Bias	
Treatment and Outcome	Studies No.	Positive Cases / Total Cases	Pooled Positive Rate, % (95%CI)	Q Value	P	I ^2^(%)	Begg’s P	Egger’s P
Surgery treatment	25	236/689	29.7(18.1–42.8)	317.31	<0.0001	92.4	0.0082	0.3828
Steroid treatment	21	383/603	78.4(65.3–89.1)	262.80	<0.0001	92.4	0.013	0.0137
Remission after steroid treatment	20	333/346	96.2(94.0–97.9)	23.76	0.2057	20	<0.0001	0.072
Relapse after steroid treatment	12	37/269	13.8(7.2–22.0)	34.91	0.0003	68.5	0.459	0.0015
Repeated steroid treatment after relapse	8	58/59	95.8(89.8–99.3)	2.37	0.9365	0	0.0786	0.7643
Remission after repeated steroid treatment	8	56/58	94.8(88.2–98.8)	4.28	0.7466	0	0.0786	0.6022

## Discussion

It has been nearly two decades since Yoshida *et al*. proposed the term autoimmune pancreatitis in 1995 [2] and AIP has now become a focus in research. Our study provided the comprehensive data of Chinese AIP patients. This study showed that the clinical features of AIP patients in China were consistent with the reports from other countries [6, 13, 17] except for the considerable proportion of pancreatic duct dilation (13.8%). Nearly one-third patients (29.7%) underwent surgeries due to suspected malignancy. The steroid regimen with an initial dose of 30 mg, 40 mg or 0.6mg/kg prednisone per day was effective in most patients with low rates of relapse and complications.

AIP is a rare disease and still a diagnostic challenge despite of increasing study in recent years. As a region with high prevalence of pancreatic disease, China would have a large amount of AIP patients. However, the concept of AIP has just been introduced in this decade and the first clinical report about Chinese AIP patients was published in 2008 [7]. In this study, we could only collect 706 patients from 26 articles, which suggested a large gap in the study of AIP between China and developed countries. Nowadays, several AIP diagnostic criteria including Japanese criteria, Mayo Clinic's HISORt criteria, Korean criteria, Asian diagnostic criteria and ICDC were proposed. Our study showed that the criteria above were adopted by different institutions in China, and 58.9% of AIP patients were diagnosed by Asian diagnostic criteria. Asian diagnostic criteria are simple and have good practicability, and may be more suitable for Chinese AIP patients due to similar ethnic background.

In ICDC, AIP is classified into two subtypes. Type 1 is recognized to be part of IgG4-related disease and type 2 is characterized by intraductal neutrophilic infiltration and no IgG4 elevation [4]. Type 2 only covered a proportion of 1% to 6% AIP patients in East Asia, while this proportion increased to 15% to 38% in Europe and North America [3, 18–20]. The present study revealed that type 2 AIP covered a proportion of 4.7% in China. This low proportion was consistent with the previous studies from Japan and Korea. Notably, type 2 AIP is much more difficult to be diagnosed because there is no specific serum marker and histological examination is always required. However, in clinical practice, an adequate specimen of the pancreas is not frequently available. In this study, 11.2% AIP patients received EUS-guided or CT-guided FNA but most of them only obtained cytology diagnosis. Several recent studies also showed the difficulty of obtaining adequate specimens for histopathological evaluation through FNA [21, 22]. From this perspective, the true prevalence of type 2 AIP in China may be underestimated. Due to the low proportion of type 2, our study mainly showed the features of type 1 AIP.

The clinical presentation of Chinese AIP was similar with previous reports from other countries [6, 13, 17]. The most common presentation were obstructive jaundice (pooled rate: 63.4%, 95%CI: 55.4%–71.0%) and abdominal symptoms (pooled rate: 62.3%, 95%CI: 52.4%–71.7%), both of which are nonspecific symptoms. Extrapancreatic lesion is an important diagnostic clue and evidence for AIP. In this study, we found that the proportions of biliary involvement, abdominal lymph nodes enlargement, swelling salivary gland, swelling lacrimal gland, retroperitoneal fibrosis, interstitial nephritis and interstitial lung disease were 65.9%, 19.7%, 12.0%, 2.0%, 3.4%, 4.6% and 3.0%, respectively. Biliary involvement, namely, IgG4-related sclerosing cholangitis is characterized by lower and upper bile duct stricture. Our study showed that the lower common bile duct stricture and hilar/intra hepatic bile ducts stricture covered 62.3% and 3.6% patients, respectively, which was similar with previous reports [23, 24]. Ulcerative colitis was more common seen in type 2 AIP than in type 1 [4]. In an international survey of AIP, ulcerative colitis was found in 15.6% (10/64) type 2 AIP patients and 1.3% (2/153) type 1 AIP patients [20]. Another study from United States showed that ulcerative colitis was present in 15.8% (3/19) with type 2 patients and 4.0% (2/50) with type 1 patients [25]. Our study also showed a low proportion of ulcerative colitis (pooled rate: 1.6%, 95%CI: 0.6%–2.9%) in Chinese AIP patients.

CT and MRI are main pancreatic parenchymal imaging modalities and play an essential role in the diagnosis of AIP. Our study showed some differences compared with ICDC. In ICDC, diffuse enlargement and segmental/focal enlargement are proposed to be typical and indeterminate findings, respectively [4]. However, in our study, 41.6% patients were classified into focal-type. The relatively high proportion of focal-type may be attributed to the fact that about one third patients obtained diagnosis of AIP after surgery and focal-type patients tends to receive surgery with suspect of malignancy, which led to potential selection bias. Moreover, pancreatic duct imaging was adopted by ICDC as an important diagnostic evidence for AIP and long and multiple strictures were recognized as the main manifestation, while dilation of the pancreatic duct was recognized as a rare manifestation to differentiate AIP from pancreatic cancer. In our study, about two-thirds patients showed stricture of pancreatic duct (pooled rate: 68.1%, 95%CI: 49.3%–84.3%) but dilatation was also found in 13.8% patients. In the previous study from our center [16], marked upstream dilatation (duct size > 5 mm) upon stricture was seen in 16.0% (4/25) patients and in another report from China [9], dilation of pancreatic duct were found in 45.5% (10/22) type 1 AIP patients. Theoretically, the pathogenesis of AIP is different with ordinary chronic pancreatitis and dilated and irregularly shaped medium-sized and large ducts are rarely found in histology examination. Therefore, further study is needed to clarify the pancreatic duct imaging manifestation of Chinese AIP patients.

It was reported that the sensitivity of IgG4 elevation varies between 44%–95% in different settings [17, 26]. In our study, the level of serum IgG4 exceeds the upper limit of normal were observed in 86.0% patients in 9 (34.6%) articles, which indicated the similar diagnostic value for Chinese AIP patients. In the other 17 (65.4%) studies, IgG4 test could not be performed and the diagnosis of AIP depended on other evidences. However, IgG4 was the only recommended serological marker for type 1 AIP in the ICDC, performing IgG4 test would improve the accuracy and simplicity of diagnosing AIP.

Steroid treatment was given to nearly 80% AIP patients in China, including many patients who received surgery for suspected malignancy. AIP is highly responsive to steroid treatment. To date, although the ICDC defines the initial dose of steroid for induction of remission as 0.6 to 1 mg/kg per day, a steroid regimen has not been standardized, and there is no consensus on the duration of induction, the tapering schedule, and the optimal dose and duration of maintenance therapy [4, 27]. In China, an initial of 30mg or 40mg prednisone per day is adopted as the most commonly used regimen and our study showed the pooled remission rate as 96.2%, which proved the excellent response of Chinese AIP patients to steroid. Relapse after steroid treatment is an important issue. According to a multicenter, international survey, the relapse rate was 35.8% (245/684) after a long-term follow-up [28]. A study from Mayo Clinic showed that 44.8% (52/116) AIP patients experienced relapse during a median follow-up of 47 months [29]. In our study, the pooled rate of relapse was only 13.8% (37/269) with the mean follow-up time ranging from 12 to 45 months. A shorter follow-up time may explain the relatively low relapse rate in China. However, a report from Japan also showed the relapse was 12.5% after a mean follow- up of 43.5 months [30]. We think different steroid regimens may be another explanation. Maintenance steroid therapy, which could decrease relapse [31], was not adopted in most reports from Western countries but was always adopted in Eastern countries [32]. From this perspective, maintenance therapy may be a better choice for patients at high risk for relapse.

## Limitations

There are several limitations of this study. First, due to the nature of this systematic review, publication bias could not be avoided and significant heterogeneity existed in many aspects. We believed this is a suboptimal and feasible option to provide an overview of AIP in China, when a national study is not available. Second, type 1 and type 2 were clearly defined in ICDC in 2011; however, not all enrolled studies adopted these criteria, so we could not exactly distinguish type 1 and type 2 AIP. However, with low proportion of type 2, our study mainly showed the features of Chinese type 1 AIP patients.

## Conclusions

In conclusion, type 1 is the predominant type of Chinese AIP patients and the clinical features, diagnostic modalities and therapeutic regimen were fundamentally similar with those in other countries. Knowledge of AIP should be more widespread to avoid unnecessary surgery.

## Supporting Information

S1 PRISMA Checklist Preferred Reporting Items for Systematic Reviews and Meta-Analyses (PRISMA) statement checklist. (DOC)

S1 File Enrolled studies except for these cited in the text. (DOC)

S2 File Detailed data and forest plots for all of the variables. (RTF)
