# Supplementary material for: Diagnosis and Treatment of Autoimmune Pancreatitis in China: A Systematic Review
Source: PLoS One. 2015 Jun 25;10(6):e0130466. doi: 10.1371/journal.pone.0130466 (PMC4481503; doi:10.1371/journal.pone.0130466)
Supplement: S1 File — (DOC) [file pone.0130466.s002.doc]

**S1 File. Enrolled studies** **except for these cited in the text.**

1. Tang HC, Liang J, Wang H, Sun LY. Clinical analysis of autoimmune pancreatitis: a report of 11 cases. Chinese Journal of Rheumatology. 2010; 14: 624 - 626.

## 2. Hu SD, Chen YR, Jia YJ, Ni EZ, Song Q, et al. Autoimmune pancreatitis: MDCT features and misdiagnosis analysis. Journal of Practical Radiology. 2012; 28:1557 -1560.

## 3. Liang L, Lu XL, Gu JY, Chen G, Rang SX, et al. Imaging characteristics of abdominal organs in autoimmune pancreatitis. Journal of Clinical Hepatology. 2012; 28:579 - 583.

4.Wu ZY. The reason analysis of autoimmune pancreatitis misdiagnosis as pancreatic cancer. Clinical Misdiagnosis and Mistherapy. 2013; 26: 26 – 28.

## 5. Chen DY, Cao HY, Chen Y, Li YM, Chen Y, et al. The clinical characteristics of 32 patients with autoimmune pancreatitis.Chinese Journal of Internal Medicine. 2014; 53: 380 – 383.

## 6.Zhang L, Huang YH, Chang H, Yao W, Wang TL, et al. Clinical features of autoimmune pancreatitis: whether associated with IgE ? Chinese Congress of Digestive Diseases.2012.

## 7. Wang XL, Yao P. Autoimmune pancreatitis clinical research in 14 cases.Journal of XinJiang Medical University. 2010; 33:1325 – 1329.

## 8. Jin WL, Zheng JL, Tie XJ. Autoimmune pancreatitis clinical diagnosis and treatment analysis in 20 cases. Medical Innovation of China. 2011; 08:154 – 155.

## 9. Xu Y, Tang W, Qian MJ. Clinical analysis on undifferentiated type of autoimmune pancreatitis in 13 cases. Modern Medicine and Health. 2013; 29: 1323 – 1324.

10. Liu B, Li G, Liu JZ. Autoimmune pancreatitis clinical diagnosis and treatment experience in 11 cases. Journal of Clinical Medicine in Practice. 2010; 14: 98 – 99.

11. Ji XL, Chen JH, Gu FY. Autoimmune pancreatitis diagnosis and treatment analysis in 16 cases. Zhejiang Clinical Medical Journal. 2013;15: 1682 – 1683.

## 12.Guo DQ, Shi JW, Dai SH. Multiple imaging comparative analysis of autoimmune pancreatitis.Journal of Practical Medical Imaging. 2012; 13: 137 – 139.

## 13. Lu Y, Huang GL, Xie XY, Xu ZF, Zheng YL, et al. (2014) Ultrasonography manifestation and differential diagnosis of autoimmune pancreatitis.Chinese Journal of Ultrasonography 23: 308 – 311.

## 14. Liu GH, Cao SC, Wang W, Li S. MRI features of autoimmune pancreatitis. Journal of Medical Imaging. 2012; 22: 2067 – 2070.

## 15. Chen Y, Ding JL, Xing W. CT and MRI Analysis of Autoimmune Pancreatitis. Chinese Journal of CT and MRI. 2013; 11: 22 – 23.

## 16. Zhu J, Fang JZ, Yang ZH, Tang WL, Li G, et al. Extrapancreatic imaging findings of autoimmune pancreatitis. Chinese Journal of Radiology. 2013; 47: 517 – 521.

## 17. Ding XM, Gao J, Ke S, Wang SH, Kong J, et al. Mis-diagnosis and mis-treatment of autoimmune pancreatitis: a clinical study of 17 cases. Chinese Journal of Digestion. 2011; 31: 221 – 225.

## 18.Lv H, Jiang WZ, Qian JM, Yang AM, Qin MW, et al. Clinical features of autoimmune pancreatitis: a case series of 16 patients. Chinese Journal of Pancreatology. 2010; 10: 155 – 158.

## 19. Wu QJ, Chen H, Lin W, Wang Q, Zheng WJ, et al. Clinical study on IgG4-related autoimmune pancreatitis. Chinese Journal of Rheumatology.2012; 16: 798 – 803.

20. Wang TL. The clinical analysis of autoimmune pancreatitis in one single center. M.Sc. Thesis, China Medical Univercity. 2013. Available: http://d.wanfangdata.com.cn/Thesis_Y2324116.aspx

21. Han WY. Clinical analysis in 21 cases of autoimmune pancreatitis and review of the Chinese reports. M.Sc. Thesis, Zhongshan Univercity. 2010. Available: http://d.wanfangdata.com.cn/Thesis_Y 1691978.aspx.

1. Kamisawa T, Kim MH, Liao WC, Liu QD, Balakrishnan V, et al. Clinical characteristic of 327 Asian patients with autoimmune pancreatitis based on Asian diagnostic criteria. Pancreas. 2011; 40: 200 – 204.
2. Wu L, Li W, Huang X, Wang Z. Clinical features and comprehensive diagnosis of autoimmune pancreatitis in China. Digestion. 2013; 88: 128-134.
