# Supplementary material for: Diagnosis and Treatment of Autoimmune Pancreatitis in China: A Systematic Review
Source: PLoS One. 2015 Jun 25;10(6):e0130466. doi: 10.1371/journal.pone.0130466 (PMC4481503; doi:10.1371/journal.pone.0130466)
Supplement: S2 File — (RTF) [file pone.0130466.s003.rtf]

S2 File. Detailed data and forest plots for all of the variables.

Supporting Fig. 1. The forest plots of the proportion of patients with obstructive jaundice.

Non-combinability of studies
Cochran Q = 116.885605 (df = 25) P < 0.0001
Moment-based estimate of between studies variance = .133692
I² (inconsistency) = 78.6% (95% CI = 68.6% to 84.3%) 

Random effects (DerSimonian-Laird)
Pooled proportion = .633766 (95% CI = .55358 to .710385)

Bias indicators
Begg-Mazumdar: Kendall's tau b = -.185185 P = 0.1932
Egger: bias = -3.385069 (95% CI = -6.14857 to -.621569) P = 0.0185

Supporting Fig. 2. The forest plots of the proportion of patients with abdominal symptoms

Non-combinability of studies
Cochran Q = 178.82319 (df = 25) P < 0.0001
Moment-based estimate of between studies variance = .22381
I² (inconsistency) = 86% (95% CI = 80.9% to 89.2%) 

Random effects (DerSimonian-Laird)
Pooled proportion = .622929 (95% CI = .523975 to .716961)

Bias indicators
Begg-Mazumdar: Kendall's tau b = -.130435 P = 0.3655
Egger: bias = -.429477 (95% CI = -4.344854 to 3.485899) P = 0.8228

Supporting Fig. 3. The forest plots of the proportion of patients with weight loss.


Non-combinability of studies
Cochran Q = 94.17662 (df = 17) P < 0.0001
Moment-based estimate of between studies variance = .163067
I² (inconsistency) = 81.9% (95% CI = 72% to 87.2%) 

Random effects (DerSimonian-Laird)
Pooled proportion = .450621 (95% CI = .347717 to .555705)

Bias indicators
Begg-Mazumdar: Kendall's tau b = .223684 P = 0.2107
Egger: bias = 5.346577 (95% CI = 2.59255 to 8.100604) P = 0.0008

Supporting Fig. 4. The forest plots of the proportion of patients with diabetes mellitus.

Non-combinability of studies
Cochran Q = 139.527877 (df = 22) P < 0.0001
Moment-based estimate of between studies variance = .204386
I² (inconsistency) = 84.2% (95% CI = 77.5% to 88.2%) 

Random effects (DerSimonian-Laird)
Pooled proportion = .268656 (95% CI = .182891 to .364201)

Bias indicators
Begg-Mazumdar: Kendall's tau b = .107356 P = 0.4918
Egger: bias = 3.09231 (95% CI = 1.873671 to 4.31095) P < 0.0001

Supporting Fig. 5. The forest plots of the proportion of asymptomatic patients.

Fixed effects (inverse variance)
Pooled proportion = .030395 (95% CI = .019201 to .044052)

Non-combinability of studies
Cochran Q = 34.234487 (df = 25) P = 0.103
Moment-based estimate of between studies variance = .013436
I² (inconsistency) = 27% (95% CI = 0% to 54.3%) 

Bias indicators
Begg-Mazumdar: Kendall's tau b = .659091 P < 0.0001
Egger: bias = .517745 (95% CI = -.131211 to 1.166701) P = 0.1127

Supporting Fig. 6. The forest plots of the proportion of lower part of the common bile duct stricture.

Non-combinability of studies
Cochran Q = 131.114195 (df = 18) P < 0.0001
Moment-based estimate of between studies variance = .254096
I² (inconsistency) = 86.3% (95% CI = 80.1% to 89.8%) 

Random effects (DerSimonian-Laird)
Pooled proportion = .622675 (95% CI = .498673 to .739018)

Bias indicators
Begg-Mazumdar: Kendall's tau b = .053098 P = 0.7792
Egger: bias = -3.886819 (95% CI = -6.646299 to -1.127339) P = 0.0086

Supporting Fig. 7. The forest plots of the proportion of hilar/intra hepatic bile ducts stricture.

Non-combinability of studies
Cochran Q = 45.655153 (df = 16) P = 0.0001
Moment-based estimate of between studies variance = .071524
I² (inconsistency) = 65% (95% CI = 34.4% to 77.8%) 

Random effects (DerSimonian-Laird)
Pooled proportion = .035961 (95% CI = .011483 to .073327)

Bias indicators
Begg-Mazumdar: Kendall's tau b = .503759 P = 0.0063
Egger: bias = .262318 (95% CI = -1.493201 to 2.017837) P = 0.7545

Supporting Fig. 8. The forest plots of the proportion of abdominal lymph nodes enlargement. 
Non-combinability of studies
Cochran Q = 190.806849 (df = 19) P < 0.0001
Moment-based estimate of between studies variance = .418656
I² (inconsistency) = 90% (95% CI = 86.5% to 92.3%) 

Random effects (DerSimonian-Laird)
Pooled proportion = .196848 (95% CI = .092022 to .329289)

Bias indicators
Begg-Mazumdar: Kendall's tau b = .555556 P = 0.0007
Egger: bias = 4.9172 (95% CI = 1.125158 to 8.709242) P = 0.0139

Supporting Fig. 9. The forest plots of the proportion of swelling of salivary gland.


Non-combinability of studies
Cochran Q = 98.383189 (df = 17) P < 0.0001
Moment-based estimate of between studies variance = .18617
I² (inconsistency) = 82.7% (95% CI = 73.5% to 87.7%) 

Random effects (DerSimonian-Laird)
Pooled proportion = .120275 (95% CI = .057109 to .202703)

Bias indicators
Begg-Mazumdar: Kendall's tau b = .695364 P < 0.0001
Egger: bias = 2.568116 (95% CI = 1.069094 to 4.067137) P = 0.0022

Supporting Fig. 10. The forest plots of the proportion of swelling of lacrimal gland.


Non-combinability of studies
Cochran Q = 32.32872 (df = 17) P = 0.0137
Moment-based estimate of between studies variance = .032287
I² (inconsistency) = 47.4% (95% CI = 0% to 68.4%) 

Random effects (DerSimonian-Laird)
Pooled proportion = .027097 (95% CI = .010419 to .051299)

Bias indicators
Begg-Mazumdar: Kendall's tau b = .84106 P < 0.0001
Egger: bias = .760744 (95% CI = .158228 to 1.36326) P = 0.0165

Supporting Fig. 11. The forest plots of the proportion of the patients with the symptom similar with Sjögren syndrome.


Non-combinability of studies
Cochran Q = 29.235679 (df = 11) P = 0.0021
Moment-based estimate of between studies variance = .070798
I² (inconsistency) = 62.4% (95% CI = 14.9% to 78.3%) 

Random effects (DerSimonian-Laird)
Pooled proportion = .088654 (95% CI = .041575 to .151164)

Bias indicators
Begg-Mazumdar: Kendall's tau b = .6 P = 0.0088
Egger: bias = 2.260232 (95% CI = .918347 to 3.602117) P = 0.0038

Supporting Fig. 12. The forest plots of the proportion of the patients with interstitial nephritis.


Non-combinability of studies
Cochran Q = 52.569122 (df = 17) P < 0.0001
Moment-based estimate of between studies variance = .073196
I² (inconsistency) = 67.7% (95% CI = 42% to 79%) 

Random effects (DerSimonian-Laird)
Pooled proportion = .046047 (95% CI = .018755 to .084615)

Bias indicators
Begg-Mazumdar: Kendall's tau b = .653061 P = 0.0003
Egger: bias = 1.040026 (95% CI = .07638 to 2.003672) P = 0.0361

Supporting Fig. 13. The forest plots of the proportion of the patients with retroperitoneal fibrosis.


Fixed effects (inverse variance)
Pooled proportion = .03414 (95% CI = .019754 to .052268)

Non-combinability of studies
Cochran Q = 16.841962 (df = 13) P = 0.2066
Moment-based estimate of between studies variance = .008996
I² (inconsistency) = 22.8% (95% CI = 0% to 58.8%) 

Bias indicators
Begg-Mazumdar: Kendall's tau b = .4 P = 0.0546
Egger: bias = .67103 (95% CI = -.274773 to 1.616833) P = 0.1481

Supporting Fig. 14. The forest plots of the proportion of the patients with interstitial lung disease.


Non-combinability of studies
Cochran Q = 36.91797 (df = 15) P = 0.0013
Moment-based estimate of between studies variance = .05008
I² (inconsistency) = 59.4% (95% CI = 18.5% to 75.3%) 

Random effects (DerSimonian-Laird)
Pooled proportion = .029957 (95% CI = .009864 to .060459)

Bias indicators
Begg-Mazumdar: Kendall's tau b = .747826 P = 0.0001
Egger: bias = .806148 (95% CI = -.124768 to 1.737065) P = 0.0844

Supporting Fig. 15. The forest plots of the proportion of ulcerative colitis


Fixed effects (inverse variance)
Pooled proportion = .015624 (95% CI = .006328 to .028962)

Non-combinability of studies
Cochran Q = 4.190977 (df = 13) P = 0.989
Moment-based estimate of between studies variance = 0
I² (inconsistency) = 0% (95% CI = 0% to 47.4%) 

Bias indicators
Begg-Mazumdar: Kendall's tau b = .383721 P = 0.0748
Egger: bias = -.061965 (95% CI = -.518632 to .394702) P = 0.7726

Supporting Fig. 16. The forest plots of the proportion of delayed enhancement


Non-combinability of studies
Cochran Q = 38.412957 (df = 12) P = 0.0001
Moment-based estimate of between studies variance = .098908
I² (inconsistency) = 68.8% (95% CI = 36.6% to 81%) 

Random effects (DerSimonian-Laird)
Pooled proportion = .943599 (95% CI = .884858 to .98232)

Bias indicators
Begg-Mazumdar: Kendall's tau b = -.87013 P < 0.0001
Egger: bias = -1.377857 (95% CI = -2.119976 to -.635739) P = 0.0018

Supporting Fig. 17. The forest plots of the proportion of rim-like enhancement


Non-combinability of studies
Cochran Q = 162.472318 (df = 17) P < 0.0001
Moment-based estimate of between studies variance = .342161
I² (inconsistency) = 89.5% (95% CI = 85.4% to 92%) 

Random effects (DerSimonian-Laird)
Pooled proportion = .626808 (95% CI = .482928 to .760057)

Bias indicators
Begg-Mazumdar: Kendall's tau = -.150327 P = 0.3686
Egger: bias = 1.547565 (95% CI = -4.337275 to 7.432406) P = 0.5849

Supporting Fig. 18. The forest plots of the proportion of calcification in parenchyma


Non-combinability of studies
Cochran Q = 39.935933 (df = 20) P = 0.0051
Moment-based estimate of between studies variance = .042127
I² (inconsistency) = 49.9% (95% CI = 5.4% to 68.6%) 

Random effects (DerSimonian-Laird)
Pooled proportion = .039093 (95% CI = .01802 to .067797)

Bias indicators
Begg-Mazumdar: Kendall's tau b = .778325 P < 0.0001
Egger: bias = .870342 (95% CI = .327754 to 1.412931) P = 0.0033

Supporting Fig. 19. The forest plots of the proportion of pancreatic pseudocysts.


Fixed effects (inverse variance)
Pooled proportion = .033623 (95% CI = .01973 to .051041)

Non-combinability of studies
Cochran Q = 17.381213 (df = 20) P = 0.6281
Moment-based estimate of between studies variance = 0
I² (inconsistency) = 0% (95% CI = 0% to 41.5%) 

Bias indicators
Begg-Mazumdar: Kendall's tau b = .36 P = 0.0303
Egger: bias = .528199 (95% CI = .028476 to 1.027922) P = 0.0394

Supporting Fig. 20. The forest plots of the proportion of stricture of pancreatic duct.

<5/14>

Non-combinability of studies
Cochran Q = 232.828899 (df = 17) P < 0.0001
Moment-based estimate of between studies variance = .63477
I² (inconsistency) = 92.7% (95% CI = 90.4% to 94.2%) 

Random effects (DerSimonian-Laird)
Pooled proportion = .681095 (95% CI = .492906 to .842798)

Bias indicators
Begg-Mazumdar: Kendall's tau = -.294118 P = 0.0813
Egger: bias = -4.894508 (95% CI = -7.921329 to -1.867687) P = 0.0035

Supporting Fig. 21. The forest plots of the proportion of upstream pancreatic duct dilatation.


Supporting Fig. 22. The forest plots of the proportion of pancreatic duct calculi.


Fixed effects (inverse variance)
Pooled proportion = .018695 (95% CI = .008519 to .03272)

Non-combinability of studies
Cochran Q = 11.969737 (df = 19) P = 0.8869
Moment-based estimate of between studies variance = 0
I² (inconsistency) = 0% (95% CI = 0% to 42.2%) 

Bias indicators
Begg-Mazumdar: Kendall's tau b = .775862 P < 0.0001
Egger: bias = .261695 (95% CI = -.159962 to .683352) P = 0.2087

Supporting Fig. 23. The forest plots of the proportion of patients with higher level of serum IgG4.


Non-combinability of studies
Cochran Q = 31.714652 (df = 8) P = 0.0001
Moment-based estimate of between studies variance = .145044
I² (inconsistency) = 74.8% (95% CI = 42.7% to 85.4%) 

Random effects (DerSimonian-Laird)
Pooled proportion = .860134 (95% CI = .74199 to .946323)

Bias indicators
Begg-Mazumdar: Kendall's tau = -.5 P = 0.0446 (low power)
Egger: bias = -2.192827 (95% CI = -4.043666 to -.341988) P = 0.0265

Supporting Fig. 24. The forest plots of the proportion of patients with higher level of serum IgG.


Non-combinability of studies
Cochran Q = 74.872281 (df = 18) P < 0.0001
Moment-based estimate of between studies variance = .149583
I² (inconsistency) = 76% (95% CI = 60.8% to 83.5%) 

Random effects (DerSimonian-Laird)
Pooled proportion = .702758 (95% CI = .606263 to .790894)

Bias indicators
Begg-Mazumdar: Kendall's tau b = -.223529 P = 0.195
Egger: bias = -1.896587 (95% CI = -6.145598 to 2.352424) P = 0.3595


Supporting Fig. 25. The forest plots of the proportion of patients with higher level of serum IgE.


.

Non-combinability of studies
Cochran Q = 37.06302 (df = 3) P < 0.0001
Moment-based estimate of between studies variance = .580281
I² (inconsistency) = 91.9% (95% CI = 82% to 95.2%) 

Random effects (DerSimonian-Laird)
Pooled proportion = .618437 (95% CI = .237294 to .929034)

Bias indicators
Begg-Mazumdar: Kendall's tau = .333333 P = 0.75 (low power)
Egger: bias = 2.213568 (95% CI = -24.600321 to 29.027457) P = 0.7564

Supporting Fig. 26. The forest plots of the proportion of patients with higher level of serum ã-globulin.
Non-combinability of studies
Cochran Q = 34.665509 (df = 11) P = 0.0003
Moment-based estimate of between studies variance = .109764
I² (inconsistency) = 68.3% (95% CI = 32.6% to 81.1%) 

Random effects (DerSimonian-Laird)
Pooled proportion = .679423 (95% CI = .56904 to .780477)

Bias indicators
Begg-Mazumdar: Kendall's tau = -.515152 P = 0.0138
Egger: bias = -5.924137 (95% CI = -9.093119 to -2.755155) P = 0.0019

Supporting Fig. 27. The forest plots of the proportion of patients with higher level of serum rheumatoid factor.


Non-combinability of studies
Cochran Q = 14.748493 (df = 5) P = 0.0115
Moment-based estimate of between studies variance = 0.110996
I² (inconsistency) = 66.1% (95% CI = 0% to 83.8%) 

Random effects (DerSimonian-Laird)
Pooled proportion = 0.356511 (95% CI = 0.207314 to 0.521593)

Bias indicators
Begg-Mazumdar: Kendall's tau = 0.466667 P = 0.2722 (low power)
Egger: bias = 3.279239 (95% CI = -0.039619 to 6.598096) P = 0.0517

Supporting Fig. 28. The forest plots of the proportion of patients with positive ANA.


Non-combinability of studies
Cochran Q = 29.212897 (df = 12) P = 0.0037
Moment-based estimate of between studies variance = 0.068216
I² (inconsistency) = 58.9% (95% CI = 8.3% to 76.3%) 

Random effects (DerSimonian-Laird)
Pooled proportion = 0.333299 (95% CI = 0.248073 to 0.424409)

Bias indicators
Begg-Mazumdar: Kendall's tau = 0.410256 P = 0.0573
Egger: bias = 3.015487 (95% CI = -0.346319 to 6.377293) P = 0.074

Supporting Fig. 29. The forest plots of the proportion of patients with higher level of serum ESR.


Fixed effects (inverse variance)
Pooled proportion = 0.594431 (95% CI = 0.504012 to 0.681731)

Non-combinability of studies
Cochran Q = 10.203992 (df = 6) P = 0.1163
Moment-based estimate of between studies variance = 0.042487
I² (inconsistency) = 41.2% (95% CI = 0% to 73.8%) 

Bias indicators
Begg-Mazumdar: Kendall's tau b = -0.5 P = 0.1668 (low power)
Egger: bias = -9.413495 (95% CI = -14.501178 to -4.325812) P = 0.0051

Supporting Fig. 30. The forest plots of the proportion of patients with higher level of serum Ca199.


Non-combinability of studies
Cochran Q = 32.157775 (df = 17) P = 0.0144
Moment-based estimate of between studies variance = 0.038548
I² (inconsistency) = 47.1% (95% CI = 0% to 68.3%) 

Random effects (DerSimonian-Laird)
Pooled proportion = 0.414898 (95% CI = 0.348794 to 0.482578)

Bias indicators
Begg-Mazumdar: Kendall's tau b = -0.013158 P = 0.9697
Egger: bias = -1.236568 (95% CI = -4.083141 to 1.610006) P = 0.3708

Supporting Fig. 31. The forest plots of the proportion of patients with marked IgG4 positive cells (>10 cells / high-power fields [HPF]).


Non-combinability of studies
Cochran Q = 14.261171 (df = 3) P = 0.0026
Moment-based estimate of between studies variance = .251365
I² (inconsistency) = 79% (95% CI = 8.3% to 90.2%) 

Random effects (DerSimonian-Laird)
Pooled proportion = .886509 (95% CI = .652192 to .996816)

Bias indicators
Begg-Mazumdar: Kendall's tau = -.333333 P = 0.3333 (low power)
Egger: bias = -1.830146 (95% CI = -7.199106 to 3.538813) P = 0.2801

Supporting Fig. 32. The forest plots of the proportion of patients receiving surgery.


Non-combinability of studies
Cochran Q = 317.309636 (df = 24) P < 0.0001
Moment-based estimate of between studies variance = .43868
I² (inconsistency) = 92.4% (95% CI = 90.5% to 93.8%) 

Random effects (DerSimonian-Laird)
Pooled proportion = .296857 (95% CI = .180947 to .427884)

Bias indicators
Begg-Mazumdar: Kendall's tau b = .38255 P = 0.0082
Egger: bias = -2.525284 (95% CI = -8.396225 to 3.345657) P = 0.3828

Supporting Fig. 33. The forest plots of the proportion of patients receiving steroid treatment.


Non-combinability of studies
Cochran Q = 262.804342 (df = 20) P < 0.0001
Moment-based estimate of between studies variance = .421559
I² (inconsistency) = 92.4% (95% CI = 90.2% to 93.9%) 

Random effects (DerSimonian-Laird)
Pooled proportion = .783912 (95% CI = .652876 to .890728)

Bias indicators
Begg-Mazumdar: Kendall's tau b = -.400966 P = 0.013
Egger: bias = -4.684733 (95% CI = -8.293126 to -1.07634) P = 0.0137

Supporting Fig. 34. The forest plots of remission rate after steroid treatment.


Fixed effects (inverse variance)
Pooled proportion = .961794 (95% CI = .939768 to .978977)

Non-combinability of studies
Cochran Q = 23.756464 (df = 19) P = 0.2057
Moment-based estimate of between studies variance = .013895
I² (inconsistency) = 20% (95% CI = 0% to 53.2%) 

Bias indicators
Begg-Mazumdar: Kendall's tau b = -.724324 P < 0.0001
Egger: bias = -.675982 (95% CI = -1.418955 to .066991) P = 0.072

Supporting Fig. 35. The forest plots of relapse rate after steroid treatment.


Non-combinability of studies
Cochran Q = 34.905844 (df = 11) P = 0.0003
Moment-based estimate of between studies variance = .094999
I² (inconsistency) = 68.5% (95% CI = 33.2% to 81.3%) 

Random effects (DerSimonian-Laird)
Pooled proportion = .137586 (95% CI = .07199 to .220139)

Bias indicators
Begg-Mazumdar: Kendall's tau = .181818 P = 0.459
Egger: bias = 2.208517 (95% CI = 1.066252 to 3.350782) P = 0.0015.

Supporting Fig. 36. The forest plots of patients receiving repeated steroid treatment after relapse.


Fixed effects (inverse variance)
Pooled proportion = .958189 (95% CI = .897646 to .992587)

Non-combinability of studies
Cochran Q = 2.370805 (df = 7) P = 0.9365
Moment-based estimate of between studies variance = 0
I² (inconsistency) = 0% (95% CI = 0% to 56.3%) 

Bias indicators
Begg-Mazumdar: Kendall's tau b = -.555556 P = 0.0786 (low power)
Egger: bias = -.054902 (95% CI = -.483098 to .373295) P = 0.7643

Supporting Fig. 37. The forest plots of remission rate after repeated steroid treatment.

Fixed effects (inverse variance)
Pooled proportion = .948123 (95% CI = .882157 to .988132)

Non-combinability of studies
Cochran Q = 4.283298 (df = 7) P = 0.7466
Moment-based estimate of between studies variance = 0
I² (inconsistency) = 0% (95% CI = 0% to 56.3%) 

Bias indicators
Begg-Mazumdar: Kendall's tau b = -.555556 P = 0.0786 (low power)
Egger: bias = -.158696 (95% CI = -.864734 to .547341) P = 0.6022
